# A Miniaturized Tri-Band Implantable Antenna for ISM/WMTS/Lower UWB/Wi-Fi Frequencies

**DOI:** 10.3390/s23156989

**Published:** 2023-08-07

**Authors:** Anupma Gupta, Vipan Kumar, Shonak Bansal, Mohammed H. Alsharif, Abu Jahid, Ho-Shin Cho

**Affiliations:** 1 Department of Interdisciplinary Courses in Engineering, Chitkara University Institute of Engineering and Technology, Chitkara University, Rajpura 140401, India; 2 Department of ECE, Sri Sai College of Engineering and Technology, Badhani, Pathankot 145001, India; 3 Department of ECE, Chandigarh University, Mohali 140413, India; 4 Department of Electrical Engineering, College of Electronics and Information Engineering, Sejong University, 209 Neungdong-ro, Gwangjin-gu, Seoul 05006, Republic of Korea; malsharif@sejong.ac.kr; 5 School of Electrical Engineering and Computer Science, University of Ottawa, 25 Templeton St., Ottawa, ON K1N 6N5, Canada; ajahi011@uottawa.ca; 6 School of Electronic and Electrical Engineering, Kyungpook National University, Daegu 41566, Republic of Korea

**Keywords:** tri-band, implantable antenna, ISM/WMTS bands, parasitic patch, asynchronous–spiral radiator, communication technologies, smart antennas

## Abstract

This study aims to design a compact antenna structure suitable for implantable devices, with a broad frequency range covering various bands such as the Industrial Scientific and Medical band (868–868.6 MHz, 902–928 MHz, 5.725–5.875 GHz), the Wireless Medical Telemetry Service (WMTS) band, a subset of the unlicensed 3.5–4.5 GHz ultra-wideband (UWB) that is free of interference, and various Wi-Fi spectra (3.6 GHz, 4.9 GHz, 5 GHz, 5.9 GHz, 6 GHz). The antenna supports both low and high frequencies for efficient data transfer and is compatible with various communication technologies. The antenna features an asynchronous-meandered radiator, a parasitic patch, and an open-ended square ring-shaped ground plane. The antenna is deployed deep inside the muscle layer of a rectangular phantom below the skin and fat layer at a depth of 7 mm for numerical simulation. Furthermore, the antenna is deployed in a cylindrical phantom and bent to check the suitability for different organs. A prototype of the antenna is created, and its reflection coefficient and radiation patterns are measured in fresh pork tissue. The proposed antenna is considered a suitable candidate for implantable technology compared to other designs reported in the literature. It can be observed that the proposed antenna in this study has the smallest volume (75 mm^3^) and widest bandwidth (181.8% for 0.86 GHz, 9.58% for 1.43 GHz, and 285.7% for the UWB subset and Wi-Fi). It also has the highest gain (−26 dBi for ISM, −14 dBi for WMTS, and −14.2 dBi for UWB subset and Wi-Fi) compared to other antennas in the literature. In addition, the SAR values for the proposed antenna are well below the safety limits prescribed by IEEE Std C95.1-1999, with SAR values of 0.409 W/Kg for 0.8 GHz, 0.534 W/Kg for 1.43 GHz, 0.529 W/Kg for 3.5 GHz, and 0.665 W/Kg for 5.5 GHz when the applied input power is 10 mW. Overall, the proposed antenna in this study demonstrates superior performance compared to existing tri-band implantable antennas in terms of size, bandwidth, gain, and SAR values.

## 1. Introduction

Due to the rapid development of low-power, miniaturized electronic devices and sensors, there is growing interest in implantable medical devices (IMDs) among researchers. IMDs have potential applications in neural stimulation, therapeutic medication, diagnosis and treatment of ailments, deep body communication, and drug delivery devices with high accuracy [1,2,3,4,5,6,7,8]. Antennas are a critical component of IMDs for wireless biotelemetry with external monitoring devices. Various wireless standards with different frequencies have been assigned by the ITU for biotelemetry, including the MICS (402–405 MHz) and ISM bands (433.05–434.79 MHz, 868–868.6 MHz, 902–928 MHz, 2.4–2.5 GHz, 5.725–5.875 GHz) for communication between in-body/on-body devices and external control and monitoring devices. The WMTS band (608–614 MHz, 1395–1400 MHz and 1427–1432 MHz) is recommended for external device control, while the interference-free subset of the unlicensed 3.5–4.5 GHz ultra-wideband (UWB) is used for high communication in WBAN.

Antenna design and performance enhancement techniques are receiving significant research interest due to the numerous factors and challenges involved in designing antennas for implantable devices as compared to free space antennas. The performance of implantable antennas is affected by the behavior of electromagnetic radiation in the highly diverse electrical properties of human tissues, which have heterogeneous permittivity and conductivity based on water content. The major challenges include the coupling of electromagnetic radiation with body tissues due to high conductivity, dielectric constant, and heterogeneous tissue layers, resulting in frequency and impedance detuning effect, signal degradation, poor gain and radiation efficiency, and tissue heating [9,10]. As a result, implant devices require antennas with higher gain, unidirectional radiation patterns, and low specific absorption rates.

Limited space for integrated circuits and antenna structures designed for implants is another issue. Consequently, antenna electrical dimensions are inversely proportional to resonance frequency and other radiation characteristics. Therefore, active techniques are needed to miniaturize the antenna while maintaining performance in practical in-body applications [11,12]. Researchers have focused on various design methodologies to optimize performance in terms of size, biocompatibility, bandwidth, tissue safety, and radiation characteristics. Miniaturization techniques include PIFA with shorting pins [13], PIFA with open-end slots [14], and spiral and meandered shape radiators [15,16,17,18].

Narrowband antennas are not ideal for implantable devices because their resonance frequency is affected by the varying electromagnetic properties of human tissues [19]. Wideband antennas that can support both lower and higher frequency bands are preferred for short and long-range communication with high-speed IoT devices [13,14,20,21,22,23]. Various techniques such as circular-patch [24], offset-fed [25], coplanar structures [26], dielectric loading [27], and reconfigurable structures [28,29] are used in the design of wideband and multi-band antennas. However, most of these structures have larger and more complex geometries, which limit their usage in BMDs. Compact and planar antenna structures are preferred for implantable devices, as larger structures require careful alignment during fabrication. Demand for UWB technology is also rising intensively in body area networks for brain and heart implants [24,30,31]. Furthermore, to understand and analyze existing state-of-the-art implant antenna structures, a brief summary of some structures is given in Table 1.

According to the above discussion of the most related works with this study, the main contribution of this study is the design of a triple-band implantable antenna with wide bandwidth performance that operates at ISM, WMTS, UWB, and Wi-Fi frequencies, making it suitable for commercial applications. The design incorporates a parasitic resonator and an asynchronous-meandered radiator with an open-ended square ring defected ground. The design is analyzed using a simplified three-layered human tissue model in CST Microwave Studio Suite. A prototype is fabricated and tested by implanting it into pork tissue, and numerical simulations are used to calculate the specific absorption rate (SAR) to determine the allowable input power. The proposed structure has incorporated the following advantages over the state-of-the-art literature.

- The proposed structure is a compact antenna with simple and planar configurations. Existing structures are designed using multiple layers, shoring pins, and PIFA technology, making the antenna thicker and more complex.
- Most Tri-band antennas are designed to resonate at ISM and WMTS bands, whereas the literature shows that the Ultra-wideband spectrum has significant advantages for Intra-body communication. Triple-band resonance was selected to make the antenna suitable for commercial body area network applications.
- The antenna has flexible characteristics, robust to structural deformation, size and shape of the tissue.
- Wider impedance bandwidth to sustain the frequency detuning effect caused by the heterogeneous body tissue effect in real-time scenarios.
- Broadside radiation characteristics with better gain for reliable communication link and low specific absorption rates.

The paper is structured as follows: Section 2 outlines the antenna’s geometry and design mechanism. The results and discussions are presented in Section 3, while the conclusion is provided in Section 4.

## 2. Geometry and Design Mechanism of Antenna

**Figure 1:**
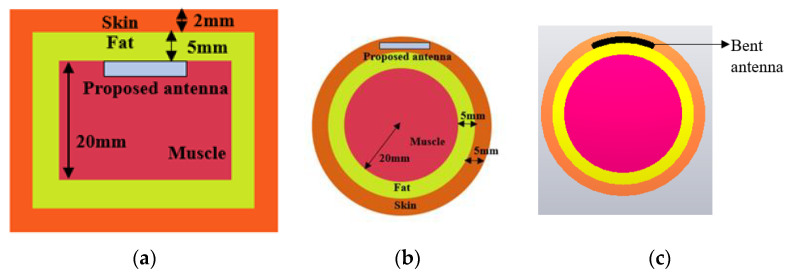
Simulation model of 3-layered tissue phantom, (**a**) rectangular phantom where antenna is implanted in muscle, (**b**) cylindrical phantom antenna implant in skin, (**c**) antenna bent across 30 mm radius.

**Figure 2:**
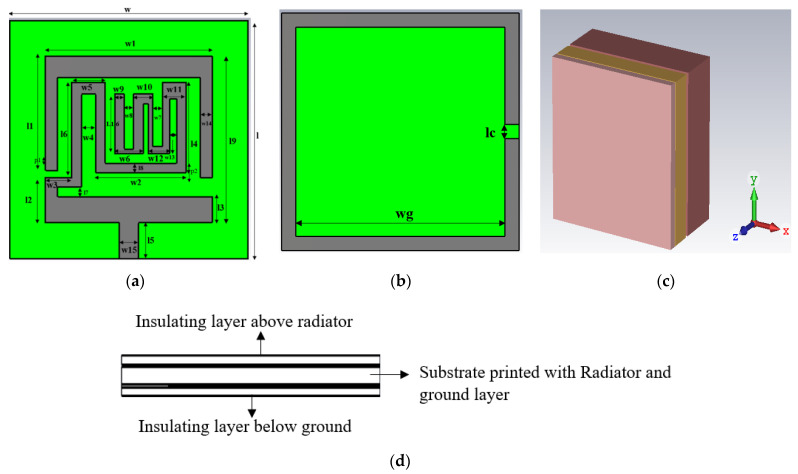
Antenna configuration: (**a**) front view, (**b**) back view, (**c**) antenna implanted in tissue, (**d**) cross-sectional view of antenna.

**Figure 3:**
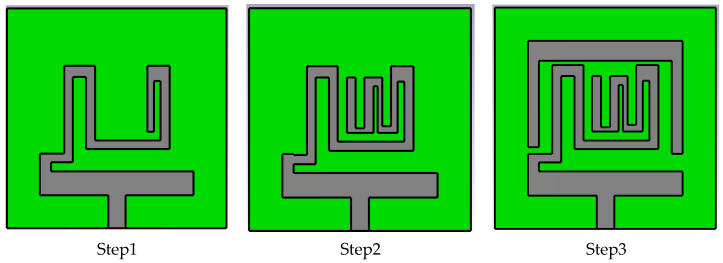
Step-wise geometry of the antenna.

- Numerical modelling of tissue: As the proposed structure is designed for implantable application, and a numerical model for the heterogeneous body tissue layers is created. It has three layers: the innermost layer is muscle tissue with a thickness of 20 mm, the middle layer is fat tissue with a 5 mm thickness, and the top layer is skin tissue with a 2 mm thickness. The human body can have different thicknesses of tissues depending upon body type, body fat or mass index, and different organs. In our model, average values for tissue thickness are considered [36,37]. Electromagnetic properties of human tissues have dispersive properties; thus, dispersive models for all three layers are taken from the CST material library for the numerical modelling of the body tissue. The planar dimensions of each layer are taken as 60 mm × 60 mm. Furthermore, to assess the sensitivity and reliability of antenna performance in practical environments, a cylindrical phantom model (radius = 30 mm, height = 40 mm) consists of skin (*ε_r_* = 38 and *σ* = 1.46 s/m), fat (*ε_r_* = 5.2 and *σ* = 0.10 s/m) and muscle (*ε_r_* = 52.7 and *σ* = 1.8 s/m) layers is designed. According to the in-body deployment location, it may be required to bend the structure for the specific tissue. Thus, antenna performance is also observed for bending. Figure 1 shows the modelled tissue phantoms, with the antenna implanted in the muscle layer (rectangular phantom) and skin layer (in cylindrical phantom) for both the flat and bent state.

- Antenna Geometry and Design Process: Configuration and geometry of the designed tri-band patch antenna is depicted in Figure 2. Table 2 contains the dimensional parameters of the proposed antenna geometry. The designed microstrip patch antenna consisted of an open-ended square loop-shaped ground plane, an asynchronous meandered radiator and a parasitic patch. The antenna structure is designed on dielectric material RO 3010 with a thickness (h) of 0.125 mm, *ε_r_* value of 10.2 and *δ* = 0.0022. The planar size of the structure is 10 mm × 10 mm, which is equivalent to (0.029 *λo* × 0.029 *λo*), where λo represents the free-space wavelength at 0.868 GHz (lowest resonance frequency). To use the antenna inside the body tissue, the radiator and ground are covered with the dielectric material layers (RO 3010). It insulates the conducting part of the antenna from lossy tissue layers and also contributes to lowering the resonance frequency. Design evaluation of the proposed structure for attaining wider bandwidth is explained in three steps. Figure 3 shows the step-wise antenna topologies used to attain the desired antenna characteristics.

**Table 2:** Dimension parameter of designed antenna.

Parameter	wg	W, l	w1	w2	w3	w4	w5	w6	w7	w8	w9	w10	l7	l9
Value (mm)	9.4	10, 10	7	4.8	1.1	0.6	1.3	1.2	0.4	0.4	0.4	1.0	0.4	7
Parameter	w11	w12	w13	w14	w15	l16	lc	l1	l2	l3	l4	l5	l6	l8
Value (mm)	1	0.9	0.3	0.5	0.8	3	0.6	4.8	1.7	1.1	4.8	1.5	4.2	0.4

To facilitate a better understanding of how to determine the structure of antennas, we will introduce a mathematical model. This model incorporates Equations (1)–(3), which represent the fundamental free space formulas. Furthermore, to analyze the impact of bio tissues, we have combined these equations with Equations (4)–(7). The details are as follows:

To achieve a lower frequency of 0.86 GHz, we initially utilized a meandered patch to miniaturize the antenna. The meandered path was incorporated to increase the length of the radiator, thereby extending the electric current path and reducing the resonance frequency [38]. Equation (1) was employed to calculate the length of the resonating path ($l_{r}$); however, we will use it to calculate the lowest resonance frequency (*f_r_*), resulting in an approximate value of 0.843 GHz, close to the simulated resonance frequency.

(1) $$l_{r}=\frac{c}{4f_{r}\sqrt{\varepsilon_{reff}}}$$

In the aforementioned equations, the symbol *c* represents the speed of light, which is approximately 3 × 10^8^ m/s. The length of the resonating path, denoted as $l_{r}$, can be calculated using Equation (2). Additionally, the effective dielectric constant of the antenna, expressed as $\varepsilon_{reff}$, is determined based on the substrate’s dielectric constant and the antenna’s dimensions, as shown in Equation (3). The value of antenna dimensional parameters used in Equation (2) are listed in Table 1.

(2) $$l_{r}\;=\;w_{1}\;+\;l_{7}\;+\;w_{3}\;+\;l_{6}\;+\;w_{5}\;+\;w_{2}\;+\;l_{4}\;+\;w_{11}\;+\;l_{16}$$

where *W* is the antenna dimension of meandered path and values are listed in Table 1. Moreover, Figure 2a shows the antenna’s meandered path.

(3) $$\varepsilon_{reff}=\frac{\varepsilon_{eq}+1}{2}+\frac{\varepsilon_{eq}-1}{2}{\left\{1+12\left(\frac{h}{w15}\right)\right\}}^{-1/2}$$

Considering that the operating environment for the antenna is a complex heterogeneous medium, the dielectric constant of the antenna varies within the layered bio tissue phantom. To account for this, we calculate the equivalent dielectric constant of the antenna, which incorporates the substrate and bio layers. This calculation is carried out using Equations (4)–(7).

(4) $$\varepsilon_{eq=\frac{d1+d2+d3+d4}{\frac{d_{1}}{\varepsilon_{r1}}+\frac{d_{2}}{\varepsilon_{r2}}+\frac{d_{3}}{\varepsilon_{r3}}+\frac{d_{4}}{\varepsilon_{r4}}}}$$

The relative permittivity of the antenna substrate and the tissue layers surrounding the antenna substrate are denoted as *ε_r_*_1_, *ε_r_*_2_, *ε_r_*_3_ and *ε_r_*_4_. Additionally, the parameters *d*_1_, *d*_2_, *d*_3_ and *d*_4_ represent the distances between the boundaries of each layer. These distances can be calculated using the methods described in references [39,40].

(5) $$d_{n}=\left\{\begin{array}{c} \frac{K\left(k_{n}\right)}{K^{’}\left(k_{n}\right)}=\begin{array}{c} \;\frac{\pi }{\ln\left[\frac{2\left(1+\sqrt{{k^{’}}_{n}}\right)}{\left(1-\sqrt{{k^{’}}_{n}}\right)}\right]}\;;\;0\;\leq \;k_{n}\;\leq \;0.707\; \end{array}\;\\ \;\frac{\ln\left[\frac{2\left(1+\sqrt{{k^{’}}_{n}}\right)}{\left(1-\sqrt{{k^{’}}_{n}}\right)}\right]}{\pi }\;;\;{0.707\leq k_{n}\leq 1}_{}^{} \end{array}\right\}$$

(6) $${K^{’}}_{n}=\sqrt{1}-{k_{n}}^{2}$$

where *n* = 1, 2…4, representing number of substrate layers; $K\left(k_{n}\right)$ and $K^{’}\left(k_{n}\right)$, are the first kind elliptical integral and its complement. The value of $k_{n}$ for the microstrip antenna can be found using Equation (7).

(7) $$k_{n}=\frac{Sinh\left(\frac{\pi W15}{2h_{n}}\right)}{Sinh\left(\frac{\pi \left(W15+2\right)}{2h_{n}}\right)}\sqrt{\frac{1-\frac{{sinh}^{2}\left(\frac{\pi \left(W15+2\right)}{2h_{n}}\right)}{{sinh}^{2}\left(\frac{\pi W}{2h_{n}}\right)}}{1-\frac{{sinh}^{2}\left(\frac{\pi W15}{2h_{n}}\right)}{{sinh}^{2}\left(\frac{\pi W}{2h_{n}}\right)}}}$$

Here, *w*15 is the feed line width, *w* is the width of the antenna, and $h_{n}$ is the height of the dielectric mediums.

**Figure 4:**
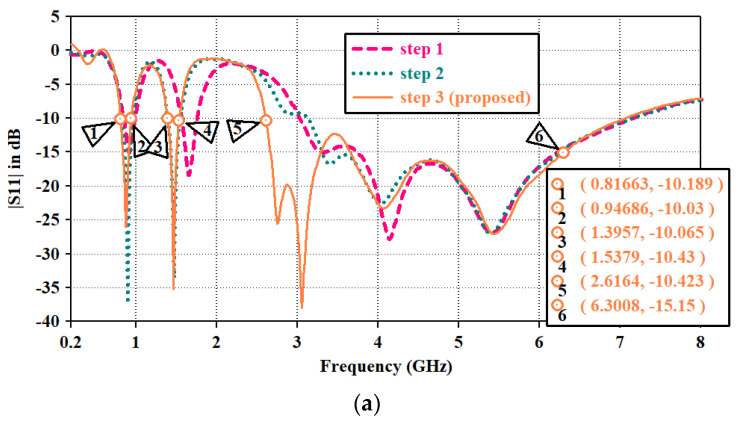

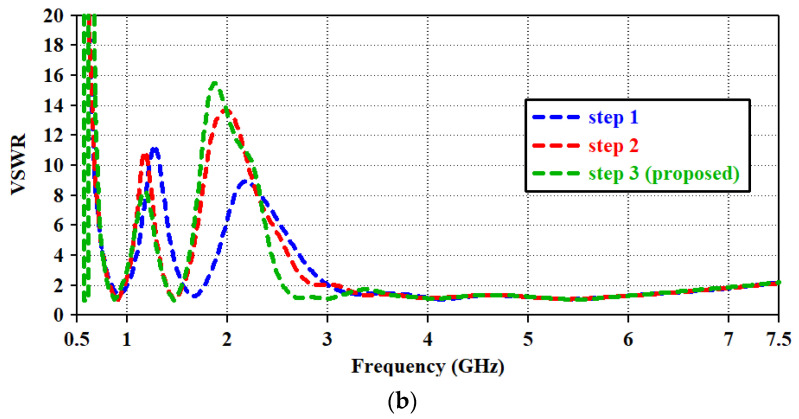
(**a**) |*S*11| plot (**b**) VSWR plot for the designed steps.

Further, in step1, an open-end slotted ground is used to improve the impedance matching. Thus, with the coupling between multiple slots of an asynchronous-meandered radiator and open-ended square loop-type ground, the multiple resonance bands are achieved in step1. The |*S*11| plot for the design steps is represented in Figure 4a. |*S*11| above 10 dB indicates the good impedance matching of the antenna, which makes the antenna efficient in terms of radiating the maximum power. *S*11 = 0 shows no power is radiated from the antenna. In the proposed design, *S*11 is above 0 at the resonance bands, making the antenna suitable in terms of radiating. To justify the step-wise impedance matching, the VSWR plot is shown in Figure 4b. A VSWR value less than 2 is desired for good impedance matching. Equations (8) and (9) show the relationship between impedance, |*S*11|, and VSWR.

(8) $$\left|S11\right|=\frac{Zl-Zo}{Zl+Zo}$$

(9) VSWR=(1+|S11|)/(1−|S11|)

here, Zl is the antenna impedance. Zo=50 ohms is characteristic of impedance.

In the first step, three resonance frequencies with bandwidths of 130 MHz (0.81–0.94 GHz), 190 MHz (1.56–1.75 GHz) and 4.03 GHz (3.03–7.06 GHz) were attained. Over the resonance bandwidth |*s*11| is above 10 due to good impedance matching, making the VSWR value less than two. It is studied from the literature that wider impedance bandwidth can be achieved by combining the resonance modes [41,42,43]. Similar technology is incorporated into this structure. *fo* is the fundamental resonance frequency at 0.8 GHz. In step1, the resonating modes are excited at *fo* = 0.8 GHz, *2fo* = 1.6 GHz, *4fo* =3.2 GHz, *5fo* = 4.0 GHz, and *7fo* = 5.6 GHz. Higher modes (4*fo* to 7*fo*) are in close proximity to each other and merged. Thus, wider impedance bandwidth from 3.03 GHz to 7.06 GHz is achieved.

It can be found that the reflection coefficient values of the lowest and middle band are 15 dB and 17 dB, respectively. Also, as middle resonance band is not at the desired frequency (1427 MHz to 1432 MHz) for the WMTS band. According to the in-body application, it has been studied in the literature that antenna impedance reduces and frequency may detune due to complex multi-layered body tissue structures. Electrical properties and depth of different tissues vary from person to person as well as for different organs. Therefore, to avoid this issue in practical situations and to tune the middle resonance frequency, the structure is modified in step 2.

**Figure 5:**
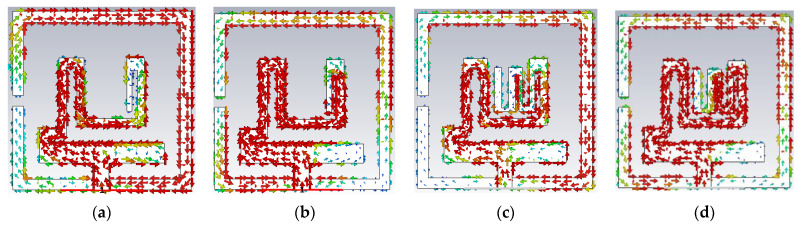
Surface current (**a**) 0.88 GHz (step1), (**b**) 1.66 GHz (step1), (**c**) 0.88 GHz (step2), (**d**) 1.46 GHz (step2).

In the second step, the length of the radiator is increased by adding a three-slot meandered element with the radiator to attain the resonance at the WMTS band. This modification has a significant impact on antenna performance. The middle band is tuned at desired range as well as the reflection coefficient value for the lower and middle bands also improved and shifted to 36 dB and 34 dB. It ensures the improvement of impedance matching. However, the upper UWB spectrum is not affected by this modification. Surface current distribution for step1 and step2 at 0.88 GHz and 1.46 GHz is shown in Figure 5. It is clear that at both frequencies, the surface current has followed the meandered path along with coupling to the ground plane. The added meandered patch has increased the effective0 length of the radiator, which helps to lower the resonance frequency from 1.66 GHz to 1.46 GHz with a bandwidth of 140 MHz (1.39–1.53 GHz). The VSWR curve for step2 in Figure 4b justifies the shifting of the resonance frequency. Impedance is matched at 1.46 GHz. Further, for the upper UWB spectrum, the lowest cutoff frequency is at the edge of the required spectrum (3.2 GHz). To enhance the impedance bandwidth toward the lower cutoff frequency, the antenna is further modified in step3. It allows the antenna to withstand frequency-detuning effects due to bio tissue.

**Figure 6:**
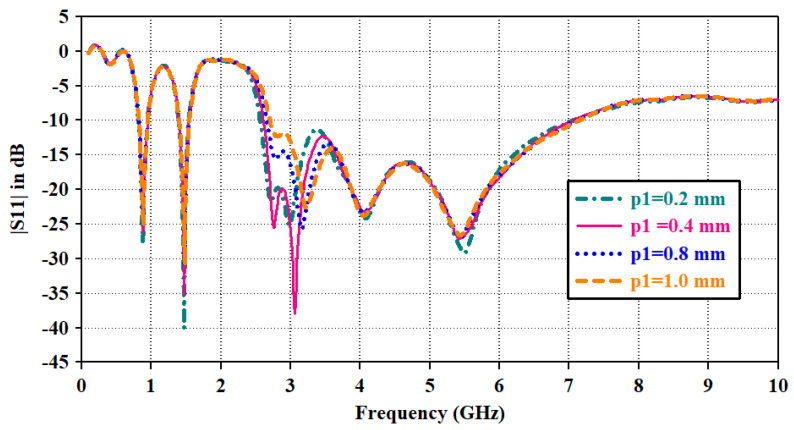
|*S*11| plot for parametric sweep for resonator length from left edge.

**Figure 7:**
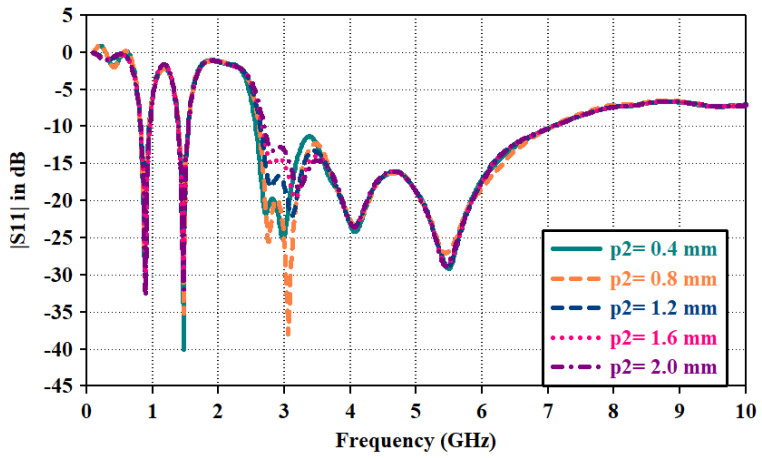
|*S*11| plot for parametric sweep for resonator length from right edge.

In the third step, an inverted U-shaped parasitic resonator is placed around the radiator. Due to capacitive coupling between the radiator and parasitic resonator, the mode at 3*fo* is excited at 2.7 GHz and merged with the resonance mode at 4*fo*; it widens the bandwidth toward a lower cut-off frequency of the upper band (|*S*11| plot of step3). In this way, the combination of various modes has contributed to the impedance bandwidth from 2.6 GHz to 6.3 GHz. The length of the parasitic resonator is optimized from both edges. Parametric analysis of the reflection coefficient for the lengths of the left and right edges is shown in Figure 6 and Figure 7, respectively. Parameter ‘p1’ varied in terms of length toward the left edge. As p1 decreases, radiator length increases, and it widens the bandwidth, whereas impedance matching at 3.5 GHz diminishes with increasing radiator length. Thus, the optimized value for ‘p1’ is maintained at 0.4 mm for wider bandwidths and proper impedance matching. Parameter ‘p2’ varied for the radiator length toward the right edge. It has a similar impact on the bandwidth, as shown in ‘p1’. Thus, the optimized value for ‘p2’ is kept at 0.8 mm.

The significance of impedance matching by adding the parasitic resonator is shown in Figure 4b. Impedance matching improves from 2.6 to 3.1 GHz, thus making the antenna suitable to radiate at the wider bandwidth. As the length of the radiator increases, mismatch losses are reducing and making the |*S*11| parameter above 0 dB in Figure 6 and Figure 7.

Consequently, a compact triple-band antenna via the embedment of open-ended square ring ground, parasitic resonator and meandered patch establishes the required features for biotelemetry systems.

**Figure 8:**
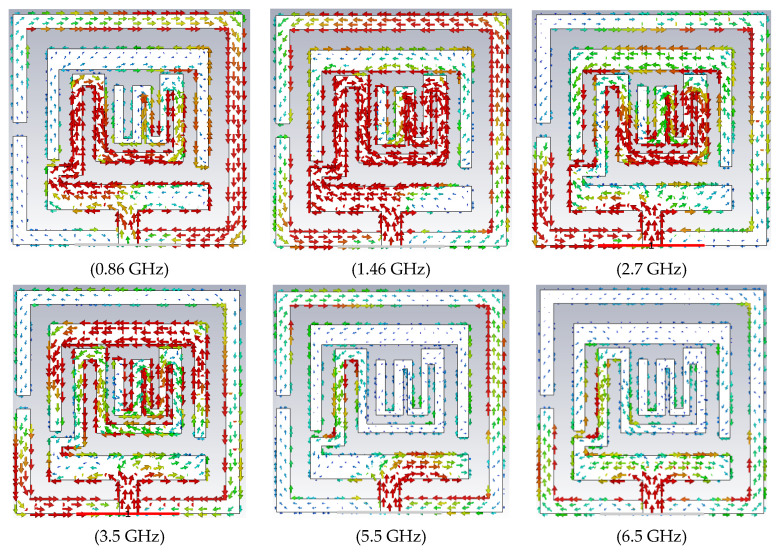
Surface current distribution at different frequencies.

Furthermore, the surface current plot for various frequencies of the antenna is shown in Figure 8. It shows that lower frequency (0.868 GHz) and middle frequency (1.43 GHz) resonances are attained due to the selected ground plane and the main radiator. It is also clear that the main resonator has excellent coupling effects on the parasitic resonator at 2.7 GHz and 3.05 GHz, which results in a wider bandwidth for the upper resonance band. Multiple current paths have excited various modes that contribute to the upper UWB spectrum.

## 3. Results and Discussions

**Figure 9:**
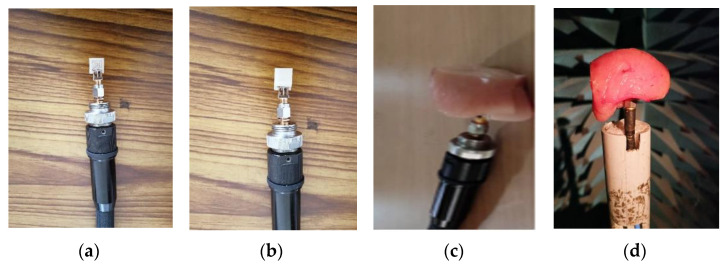
Photographs during measurement: (**a**) antenna without superstrate, (**b**) with superstrate, (**c**) antenna in animal tissue, (**d**) antenna in anechoic chamber.

**Figure 10:**
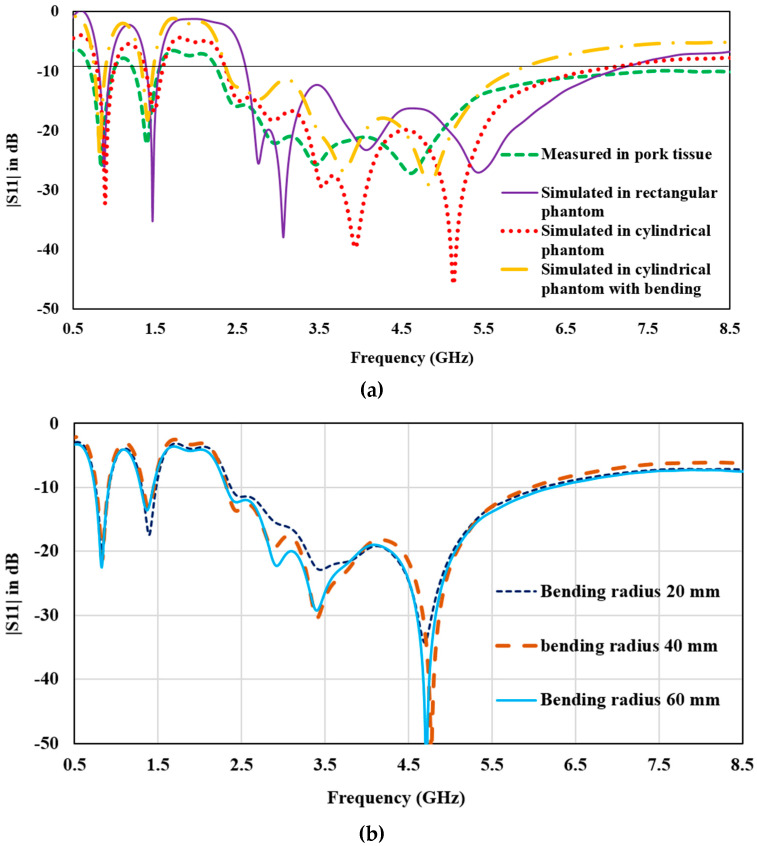
(**a**) |*S*11| plot for simulated and measured values, (**b**) |*S*11| plot for different bending radii and size of tissue.

To ensure the accuracy of the simulated results, an antenna prototype was fabricated, and its performance parameters were measured. The antenna was inserted into animal tissue for implant application, and images of the prototype and measurement setup can be found in Figure 9. Similar variations in simulation and measurement setup are shown in Figure 10 for a comparison of the measured reflection coefficients with the simulated (in rectangular and cylindrical phantom and bent state). It is practically difficult to test the antenna in real human tissue; pork is a suitable option to test antenna performance.

In the numerical simulation, the antenna is placed within the muscle layer and is effectively isolated from the surrounding tissue by the dielectric layers positioned above and below it. Among the skin and fat tissues, the muscle layer possesses the maximum implant depth, highest dielectric constant (52.7), and conductivity (1.74 S/m). As a result, the muscle tissue has the most significant influence on the antenna’s performance. To achieve a good agreement between the simulated and measured results, pork was employed as it exhibits tissue-equivalent properties. This choice of material enhances the alignment between the simulated and measured outcomes. It is worth noting that variations in simulation and measurement setups have been observed in the existing literature [13,14,22,26]. The antenna demonstrates an overlapping simulated and measured −10 dB impedance bandwidth of 120 MHz (820–940 MHz), 140 MHz (1.39–1.53 GHz), and 4.2 GHz (2.6–6.8 GHz). The measured bandwidth is slightly broader than the simulated bandwidth due to losses arising from the connector and tissue characteristics.

In order to analyze the stability of antenna performance against the shape of the implant tissue, the antenna is deployed in the skin layer of the cylindrical phantom and numerically simulated; further, the antenna is also bent along the *x*-axis, as shown in Figure 1. In comparison to the simulated results of the cylindrical and rectangular phantom, it can be observed that the reflection coefficient of the middle band shifts upward (from −35 dB to −17 dB) and the upper band shifts downwards (from −20 dB to −40 dB). This is probably due to the variation in implant depth and variation in the surface current distribution due to internal reflection and refraction caused by the fat and muscle layer below the antenna in the cylindrical phantom. Fat has a very low dielectric constant as compared to muscle tissue. According to reflection theory, at the interference of two dielectric constants, reflection and refraction take place; thus, changing the reflection coefficient. Still, the antenna impedance bandwidth for three bands is stable. On bending the antenna, lower and upper band have stable resonance, whereas the impedance bandwidth for the upper band slightly reduces toward the higher cut-off frequency. In a bent state, the bandwidth for the upper band ranges from 2.6 GHz to 5.6 GHz. This is due to the starching in the gaps of the meandered slots of the radiator. Moreover, the size of the human tissue varies from person to person; thus, the bending radii can affect the antenna performance. Thus, the thickness of the cylindrical phantom varied, and the antenna is bent for radii of 20 mm, 40 mm, and 60 mm. Figure 10b shows the plot for the antenna reflection coefficient at different bending radii. The proposed structure has a stable frequency spectrum and impedance matching. With increasing bending radius, the size of the antenna is also increased, which also ensures that the antenna has robust characteristics for varying tissue sizes.

For all of the operating conditions, the antenna has covered the bandwidth for various valuable communication standards, including ISM bands (868–868.6 MHz, 902–928 MHz, 5.725–5.875 GHz), WMTS band (1427–1432 MHz), and interference-free subset of the unlicensed 3.5–4.5 GHz ultra-wideband (UWB), as well as Wi-Fi frequencies at 3.6/4.9/5/5.9/6 GHz. These findings demonstrate the potential of the antenna for a wide range of communication applications.

**Figure 11:**
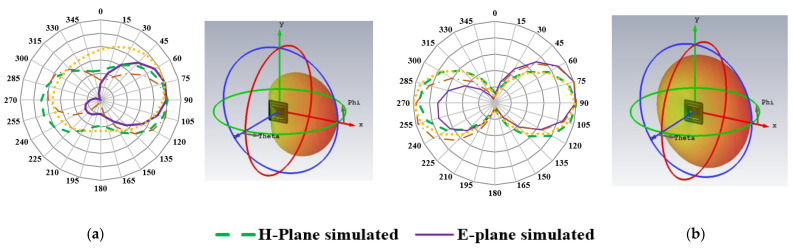

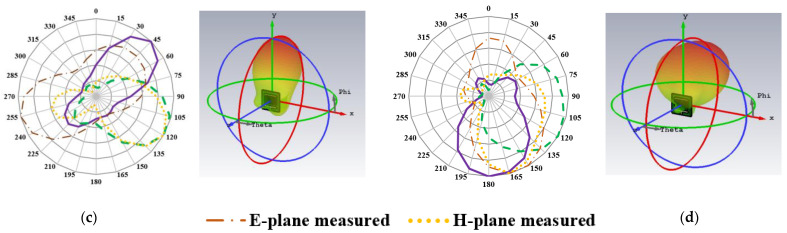
Simulated and measured 2-D and simulated 3-D radiation plots (**a**) at 0.86 GHz, (**b**) at 1.46 GHz, (**c**) at 3.5 GHz, (**d**) 5.5 GHz.

The performance of an antenna designed for in-body communication must be carefully evaluated to ensure reliable communication links and avoid any harmful effects on body tissues. One key factor in determining the effectiveness of such an antenna is its radiation pattern. To this end, Figure 11 presents the normalized 2-D radiation pattern of the proposed antenna for simulated in rectangular phantom, measured in animal tissue, along with simulated 3-D radiation plots. The measured and simulated patterns are in good agreement, indicating that the proposed antenna can effectively communicate with external devices without causing any adverse effects on body tissues. The radiation pattern of the proposed antenna at different frequencies is also examined in Figure 11. For both the simulated and measured curves, at 0.86 GHz, a broadside radiation pattern oriented towards the body surface is achieved for both the E-plane and H-plane. At 1.43 GHz, 3.5 GHz, and 5.5 GHz, unidirectional radiation patterns are attained. Measured patterns reveal larger side lobes and slight differences in the that is probably due to losses of pork tissue.

**Figure 12:**
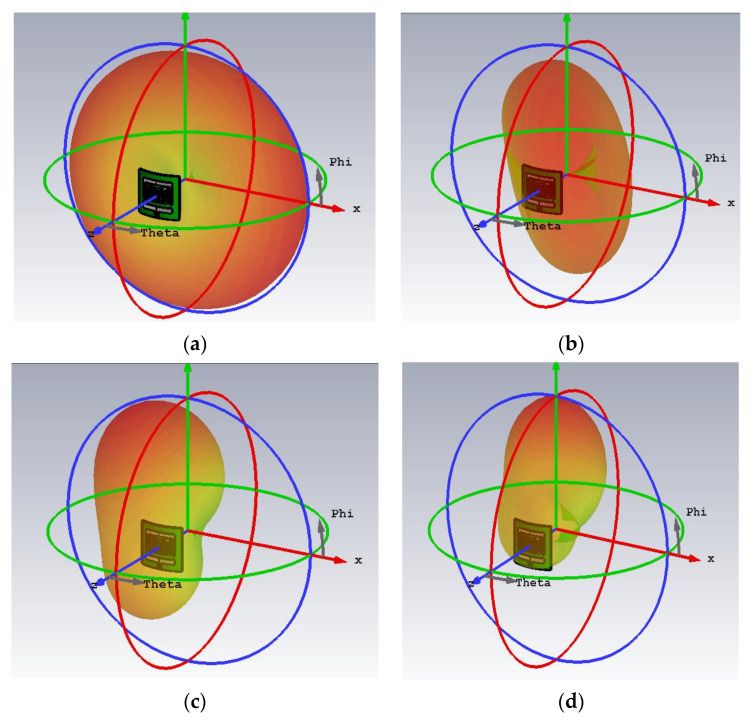
3-D radiation pattern in bent state (**a**) at 0.86 GHz, (**b**) at 1.46 GHz, (**c**) at 3.5 GHz, (**d**) 5.5 GHz.

Figure 12 presents the three-dimensional radiation pattern of the antenna when subjected to structural deformation. Similar radiation characteristics are achieved for both the flat and bent structures. It is important to note that back lobes in the radiation pattern may cause a heating effect in body tissues, so it is necessary to achieve radiation patterns that eliminate this effect. The patterns achieved by the proposed antenna are well-suited for in-body applications and provide a reliable communication link with external devices.

**Figure 13:**
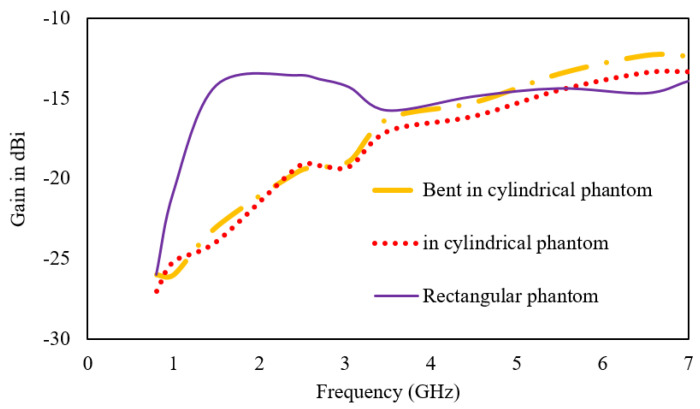
Plot for gain.

**Figure 14:**
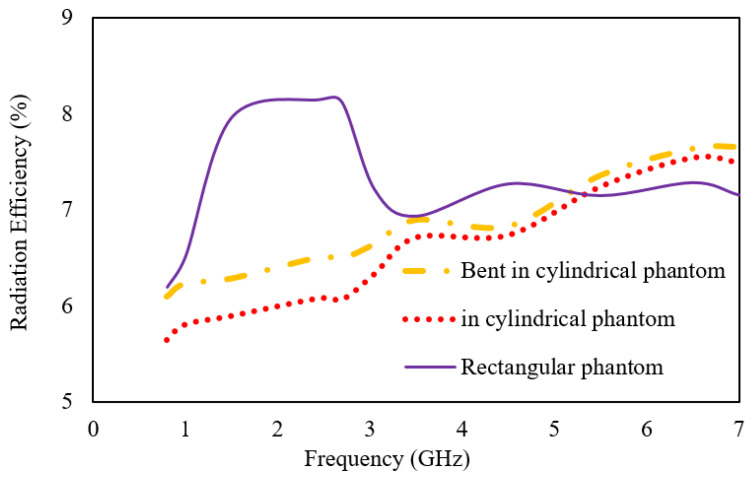
Plot for radiation efficiency.

Figure 13 and Figure 14 provide further insight into the performance of the proposed antenna by presenting the gain and radiation efficiency plots over the operating frequencies. The realized gain of the antenna at 0.8 GHz is −26 dBi; at 1.43 GHz, it is −14 dBi; at 3.5 GHz, it is −16 dBi; and at 5.5 GHz, it is −14.2 dBi. The radiation efficiency of the antenna at 0.8 GHz is 6%; at 1.43 GHz, it is 7.97%; at 3.5 GHz, it is 6.94%; and at 5.5 GHz, it is 7.15%.

When the antenna is placed below the skin layer in the cylindrical phantom and bent across the radius of 30 mm, both the gain and efficiency reduce. The middle band has the maximum deviation of 1.67% in efficiency and 9 dB in gain. These values provide important information for determining the overall performance of the antenna and its suitability for various communication applications.

In sum, the proposed antenna exhibits a radiation pattern that is well-suited for in-body communication applications and is validated through both experimental and numerical simulations. The gain and radiation efficiency plots provide further evidence of the antenna’s effectiveness across different frequencies. These findings represent a significant contribution to the development of implantable devices and pave the way for further advancements in this field.

**Figure 15:**
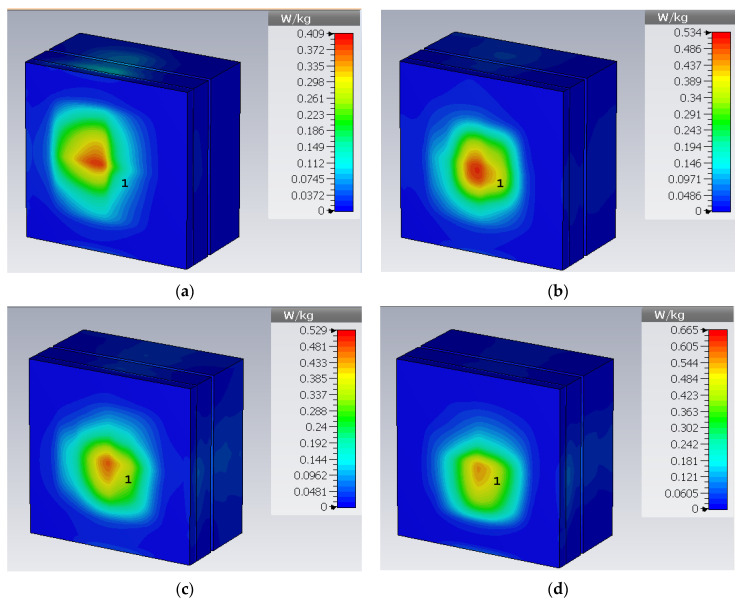
SAR plot at (**a**) 0.86 GHz, (**b**) 1.43 GHz, (**c**) 3.5 GHz, (**d**) 5.5 GHz.

**Figure 16:**
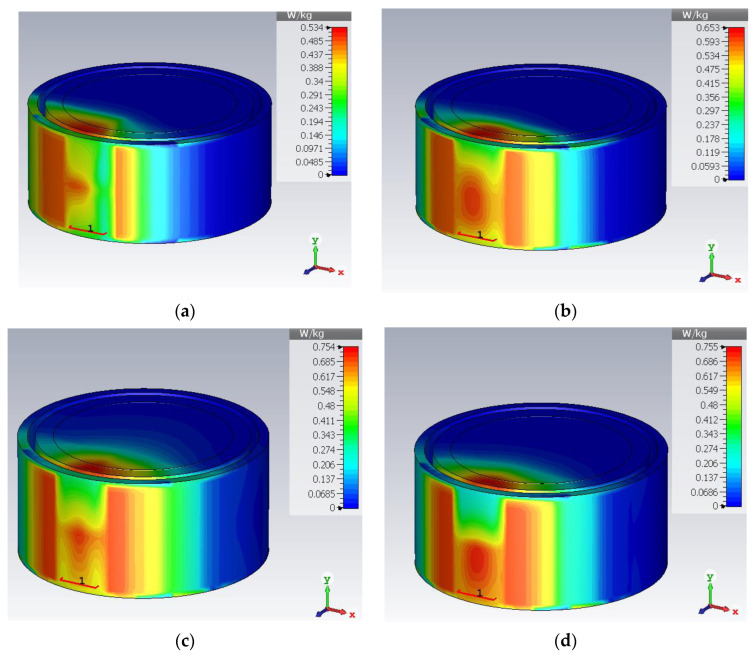
SAR plot in bent state at (**a**) 0.86 GHz, (**b**) 1.43 GHz, (**c**) 3.5 GHz, (**d**) 5.5 GHz.

The specific absorption rate is a safety parameter used to measure the amount of heat absorbed by the tissue. The IEEE Std C95.1-1999 sets a SAR limit of 1.6 W/Kg over 1 g of tissue. Figure 15 and Figure 16 present the rectangular phantom and cylindrical phantom in bent states at different frequencies. When an input power of 10 mW is applied, the SAR values are 0.409 W/Kg for 0.8 GHz, 0.534 W/Kg for 1.43 GHz, 0.529 W/Kg for 3.5 GHz, and 0.665 for 5.5 GHz. SAR value in a bent state inside a cylindrical phantom is 0.534 W/Kg for 0.8 GHz, 0.653 W/Kg for 1.43 GHz, 0.754 W/Kg for 3.5 GHz, and 0.755 for 5.5 GHz.

Table 3 presents the summary of the antenna when implanted in the muscle layer of a rectangular heterogeneous tissue phantom, the skin layer of a cylindrical-shaped tissue phantom and when subjected to structural deformation by bending along the *x*-axis at the radius of 30 mm. This analysis confirms the reliability of using the antenna inside different body organs. The antenna shows stable resonance frequencies with a slight reductions in bandwidth at the upper band. Gain and efficiency parameters are also stable over the entire bandwidth.

Finally, Table 4 presents a comparison of various tri-band implantable antennas in terms of their operating frequencies, volume, impedance bandwidth, gain, and specific absorption rate (SAR) for 1 g tissue with 1 W power. The antennas are compared based on their performance parameters, and it can be observed that the proposed antenna in this study has the smallest volume (75 mm^3^) and widest bandwidth (181.8% for 0.86 GHz, 9.58% for 1.43 GHz, and 285.7% for UWB subset and Wi-Fi). It also has the highest gain (−26 dBi for ISM, −14 dBi for WMTS, and −14.2 dBi for UWB subset and Wi-Fi) compared to other antennas in the literature. In addition, the SAR values for the proposed antenna are well below the safety limits prescribed by IEEE Std C95.1-1999, with SAR values of 0.409 W/Kg for 0.8 GHz, 0.534 W/Kg for 1.43 GHz, 0.529 W/Kg for 3.5 GHz, and 0.665 W/Kg for 5.5 GHz when the applied input power is 10 mW. Overall, the proposed antenna in this study demonstrates superior performance compared to existing tri-band implantable antennas in terms of size, bandwidth, gain, and SAR values.

## 4. Conclusions

This study presented a compact and efficient implantable antenna operating at triple bands. The proposed antenna employs a combination of a multi-open-end slotted meandered radiator, a parasitic patch, and a square ring-shaped ground to excite multiple resonant modes for various biotelemetry applications. The proposed antenna in this study has several notable advantages over other antennas in the literature. It has the smallest volume of 75 mm^3^ and the widest bandwidth of 181.8% for 0.86 GHz, 9.58% for 1.43 GHz, and 285.7% for the UWB subset and Wi-Fi. It also has the highest gain, with values of −26 dBi for ISM, −14 dBi for WMTS, and −14.2 dBi for the UWB subset and Wi-Fi. Furthermore, the SAR values of the proposed antenna are well within the safety limits prescribed by IEEE Std C95.1-1999, with SAR values of 0.409 W/Kg for 0.8 GHz, 0.534 W/Kg for 1.43 GHz, 0.529 W/Kg for 3.5 GHz, and 0.665 W/Kg for 5.5 GHz when the applied input power is 10 mW. Overall, this antenna demonstrates superior performance in terms of size, bandwidth, gain, structural deformation, and SAR values, making it a promising candidate for implantable medical devices.

## Figures and Tables

**Table 1:** Summary of state-of-the-art literature for implant antenna technology.

Refs.	Frequency	Technique	Performance Enhancement Factor	Flexible	Gain	Implant Tissue	Bandwidth
[32] 2023	1.4, 2.4 GHz	Meandered stack patch	Miniaturization 10 × 10 × 0.635 mm^3^	No	−37, −21	Homogeneous Skin/4 mm	3.57, 6.37%
[33] 2023	402.5 MHz, 2.45, 2.95 GHz	Capacitive loading	To achieve multiple bands and miniaturization 20 × 12 × 2.2 mm^3^	No	−29.7, −3.1, −7.3	Three layered tissue, implanted in muscle at 8 mm	13.98 MHz, 0.3 GHz, 0.62 GHz
[34] 2023	2.45 GHz	Stacked patch	For gain enhancement	No	−18.41	Scalp implant	100 MHz
[35] 2022	0.79 to 3.83 GHz (UWB)	Shorting pin and defected ground	For bandwidth enhancement	No	−20.22	Homogeneous skin tissue and heart	3040 MHz
[24] 2023	3.0 to 5.0 GHz (UWB)	Defected ground and adding of insulating layer	Gain and bandwidth enhancement	No	−18.6 dBi	Brain implant in Dura layer	2000 MHz
[5] 2022	403 MHz	PIFA	Miniaturized 10 × 10 × 1.905 mm^3^	No	−18.8 dBi	Three-layered phantom, implant in muscle tissue	29 MHz
[19] 2022	2.45 GHz	CPW fed with defected ground	miniaturization (585.55 mm^3^)	No	−15 dBc	Three-layered phantom, implant in muscle tissue at 4 mm	1100 MHz
This work	0.86 GHz 1.43 GHz 2.6–6.8 GHz	Patch with parasitic resonator	Multiband structure with wider impedance bandwidth and robust in-body performance	Yes	−26 dBi −14 dBi −16 dBi	Three-layered structure, implant in muscle (rectangle), skin (cylinder)	120 140 4200

**Table 3:** Summary of antenna performance under different operating conditions during simulation.

Phntom	Impant Layer	Operating Frequency Range (GHz)	Bandwidth	Resonance Frequency (GHz)	|*S*11| in dB	Gain (dBi)	Effifiency (%)	SAR (W/kg.) for 10 mW
3-Layered rectangular	Muscle	0.82–0.94	120 MHz	0.83	−27.66	−26	6.2	0.409
		1.39–1.53	140 MHz	1.43	−35.18	−14	7.97	0.534
		2.6–6.8	4.2 GHz	3.5 5.5	−12.56 −25.88	−16 −14.32	6.94 7.15	0.529 0.665
3-layered cylindrical	Skin	0.82–0.942	118 MHz	0.83	−32.20	−27	5.65	-
		1.41–1.54	139 MHz	1.43	−17.68	−24	5.9	-
		2.45–6.85	4.4 GHz	3.5 5.5	−29.42 −15.38	−17.04 −14.48	6.71 7.24	-
3-layered cylindrical (antenna in bent state)	Skin	0.82–0.936	116 MHz	0.83	−24.66	−26	6.09	0.534
		1.38–1.50	120 MHz	1.43	−18.23	−23	6.28	0.653
		2.45–5.77	3.32 GHz	3.5 5.5	−21.52 −21.68	−16.26 −13.46	6.89 7.36	0.754 0.755

**Table 4:** Comparison of antenna parameters with existing implantable antennas.

Ref.	Frequency GHz	Volume (mm^3^)	Impedance Bandwidth	Gain (dBi)	SAR (W/Kg) for 1 g Tissue with 1 W Power
[13]	0.402 (MICS)	197.04	23.13%	−33	241
	1.430 (WMTS)		14.93%	−21.9	269
	2.450 (ISM)		18.12%	−19.6	290
[20]	0.400 (MICS)	201.6	120 MHz	−35	94
	1.450 (ISM)		320 MHz	−10	296
	5.780 (Wi-Fi)		1200 MHz	−16	572
[14]	0.915 (ISM)	21	8.7%	−26.4	380
	1.900 (MF)		8.2%	−23	388
	2.450 (ISM)		7.3%	−20.47	363
[21]	0.433 (MICS)	255	18.94%	−33.76	229
	0.915 (ISM)		17.04%	−16.8	264
	2.450 (ISM)		30.96%	−21.2	245
[22]	0.402 (MICS)	405.9	-	−43.6	99.5
	0.902 (ISM)		-	−25.8	207.8
	2.400 (ISM)		-	−20.1	272
This work	0.86 (ISM)	75	120 MHz (181.8%)	−26	0.409 (for 10 mW)
	1.430 (WMTS)		140 MHz (9.58%)	−14	0.534 (for 10 mW)
	2.6–6.8 (UWB subset and Wi-Fi)		4.2 GHz (285.7%)	−16 (3.5 GHz) −14.2 (5.5 GHz)	0.529 (for 10 mW) 0.665 (for 10 mW)

## Data Availability

Not Applicable.
